# Supplementary material for: Elevated temperature and CO2 strongly affect the growth strategies of soil bacteria
Source: Nat Commun. 2023 Jan 24;14:391. doi: 10.1038/s41467-023-36086-y (PMC9873651; doi:10.1038/s41467-023-36086-y)
Supplement: Supplementary file 1 — Supplementary Information [file 41467_2023_36086_MOESM1_ESM.pdf]

Supplementary Information for

- **Elevated temperature and CO_2 strongly affect the growth strategies of soil bacteria**

Yang Ruan, Yakov Kuzyakov, Xiaoyu Liu, Xuhui Zhang, Qicheng Xu, Junjie Guo, Shiwei Guo, Qirong Shen, Yunfeng Yang, Ning Ling*

*Corresponding author. Email: nling@njau.edu.cn

This PDF file includes:

Supplementary Table 1
Supplementary Fig. 1 to 7

Supplementary Table 1 Phylogenetic indices of three ecological strategies across the treatments

Field treatment	Strategy	Num taxa	Phylogenetic dispersion	Blomberg's K	Pagel's λ
Contr	Rapid	107	**0.157** (<0.001)	**0.181** (0.001)	**0.623** (<0.001)
	Intermediate	453	**0.834** (<0.001)	**0.113** (0.002)	**0.139** (<0.001)
	Slow	190	**0.850** (<0.001)	0.094 (0.130)	**0.144** (<0.001)
eT	Rapid	184	**0.402** (<0.001)	**0.142** (0.001)	**0.491** (<0.001)
	Intermediate	675	**0.618** (<0.001)	**0.129** (0.001)	**0.437** (<0.001)
	Slow	14	1.064 (0.681)	0.083 (0.704)	0.011 (0.133)
eCO_2	Rapid	159	**0.509** (<0.001)	**0.129** (0.001)	**0.407** (<0.001)
	Intermediate	684	**0.784** (<0.001)	**0.109** (0.002)	**0.238** (<0.001)
	Slow	13	0.955 (0.370)	0.087 (0.566)	<0.001 (1.000)
$eTeCO_2$	Rapid	142	**0.485** (<0.001)	**0.131** (0.002)	**0.406** (<0.001)
	Intermediate	241	**0.842** (<0.001)	**0.101** (0.018)	**0.116** (0.008)
	Slow	287	**0.771** (<0.001)	**0.109** (0.004)	**0.244** (<0.001)

Contr represents the ambient temperature and CO_2 concentration; eT represents warming only; eCO_2 represents elevated CO_2 concentration only; and $eTeCO_2$ represents combined warming and CO_2 enrichment. Num taxa: number of taxa. A permutation test was used for data analysis and the *p* values were calculated with 1000 permutations based on trait prevalence and phylogeny. Values in bold: $p < 0.05$. The number in parentheses is the *p*-value.

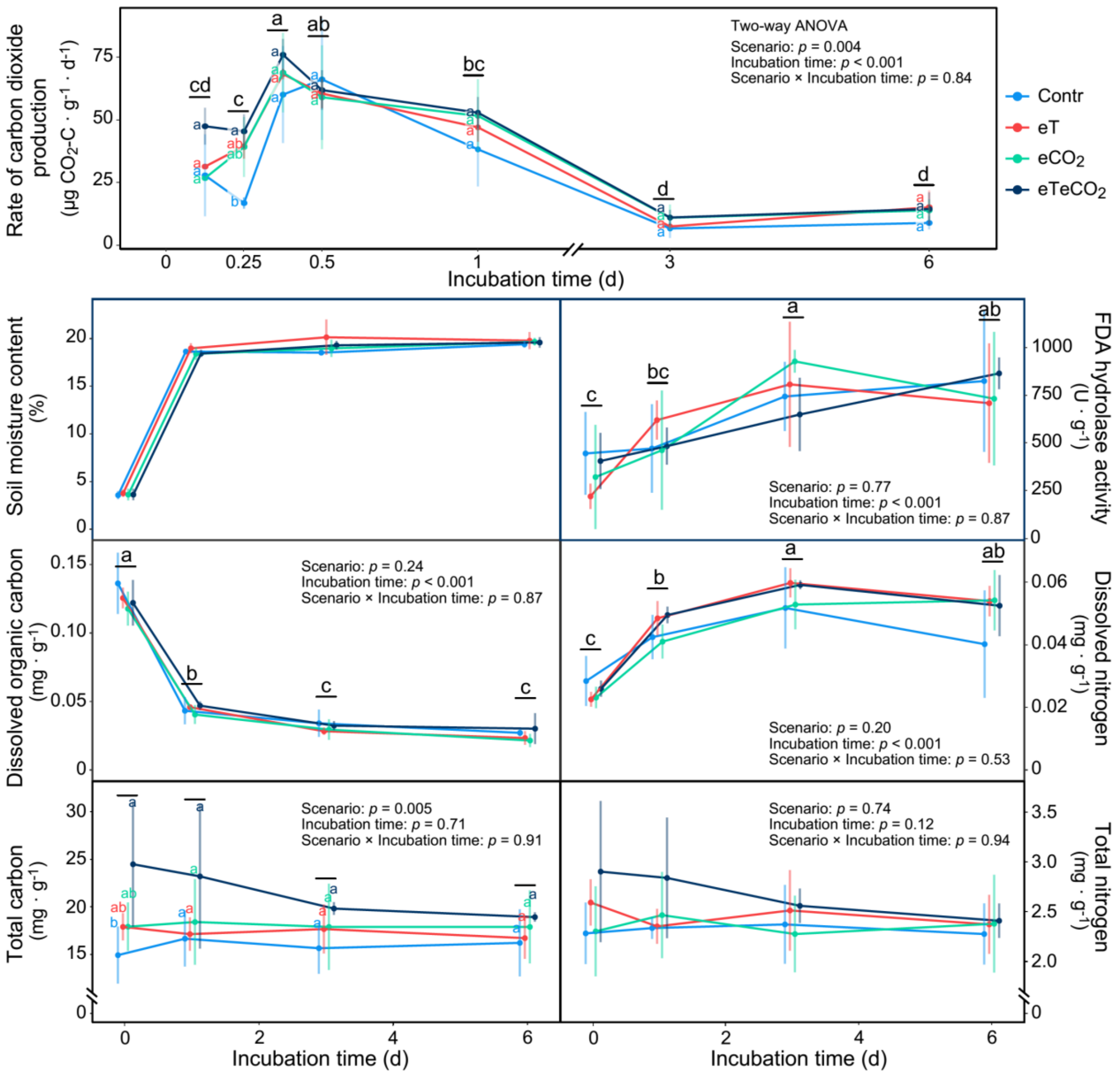

Supplementary Fig. 1

Dynamics of soil biochemical characteristics during incubation. The data of CO_2 production rates are from 7 incubation time points, i.e., 0.125 d (3 h), 0.25 d (6 h), 0.375 d (9 h), 0.5 d (12 h), 1 d, 3 d, and 6 d after water addition. Letters (black lowercase) represent significant differences between sampling times, while the colored lowercase letters indicate significant differences between climate scenarios at the same time point. The data of other measured characteristics are from 4 different incubation time points, i.e., before water addition (prewet soil), incubations for 1 d, 3 d, and 6 d, with destructive sampling. Data are presented as mean values ± SD (n = 3 biologically independent samples). ANOVA with two-sided Tukey's multiple comparisons was used for the statistical analysis. Note that the soil for these analyses was sampled in July 2022. Contr represents the ambient temperature and CO_2 concentration; eT represents warming only; eCO_2 represents elevated CO_2 concentration only; and $eTeCO_2$ represents combined warming and CO_2 enrichment.

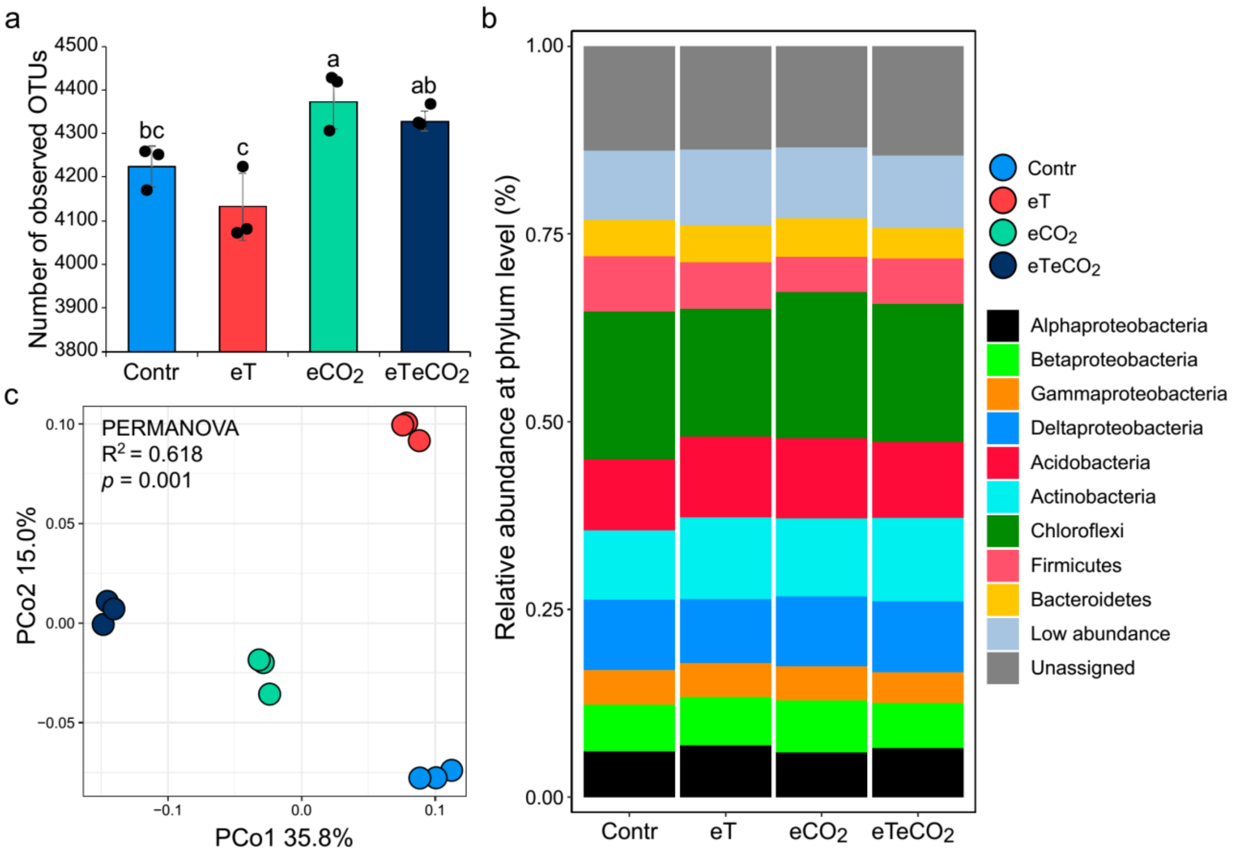

Supplementary Fig. 2

Taxonomic composition and community structure of bacterial communities at initial incubation time (0 d). Number of observed OTUs (**a**) represented the α-diversity of simulated climate change treatments: ambient temperature and CO_2 concentration (Contr), elevated temperature of canopy air by +2 °C (eT), elevated CO_2 concentration up to 500 ppm (eCO_2), and combined CO_2 enrichment and warming treatment ($eTeCO_2$). Data are presented as mean values ± SD (n = 3 biologically independent samples). ANOVA with two-sided Tukey's multiple comparisons was used for the statistical analysis. Letters: significant differences between treatments ($p < 0.05$). Taxonomic compositions of the bacterial community are presented at the phylum/class level (**b**) and principal coordinate analysis (PCoA) plot depicting the Bray–Curtis distance among four treatments (**c**). Significance of bacterial community dissimilarities among different plant microhabitats are based on Permutational multivariate analysis of variance (PERMANOVA). Contr represents the ambient temperature and CO_2 concentration; eT represents warming only; eCO_2 represents elevated CO_2 concentration only; and $eTeCO_2$ represents combined warming and CO_2 enrichment.

.

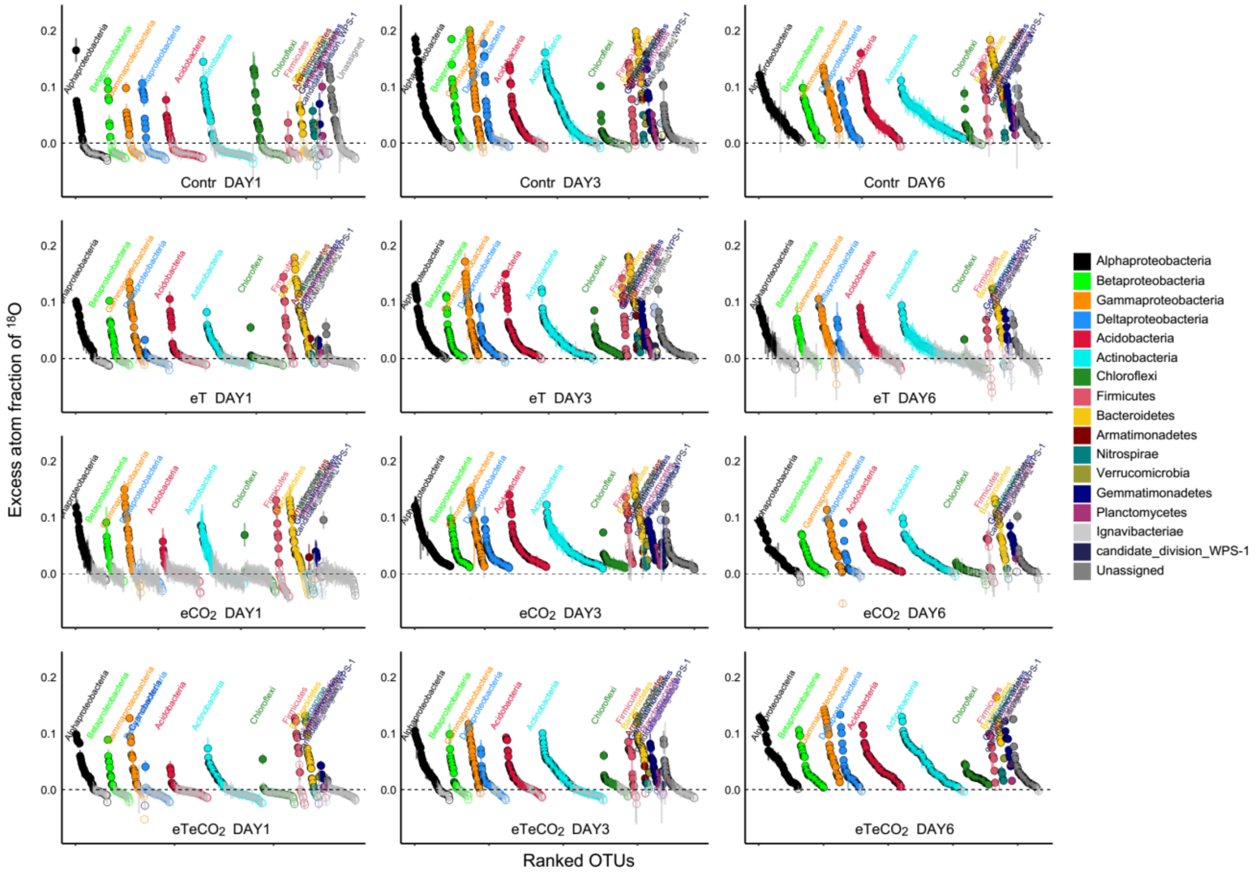

Supplementary Fig. 3

Species-specific shifts of ^{18}O excess atom fraction (EAF-^{18}O). Points are presented as mean values and bars are presented as 95% confidence intervals (CIs) of OTUs (*n* = 3 biologically independent samples). Each circle represents an OTU and was colored by phylum. The open circles with gray bars represent OTUs with 95% CI intersected with zero (indicating no significant ^{18}O enrichment); Closed circles represent the OTUs with significantly enriched ^{18}O, whose 95% CIs were not zero (i.e., the OTUs had detectable growth). Contr represents the ambient temperature and CO_2 concentration; eT represents warming only; eCO_2 represents elevated CO_2 concentration only; and $eTeCO_2$ represents combined warming and CO_2 enrichment.

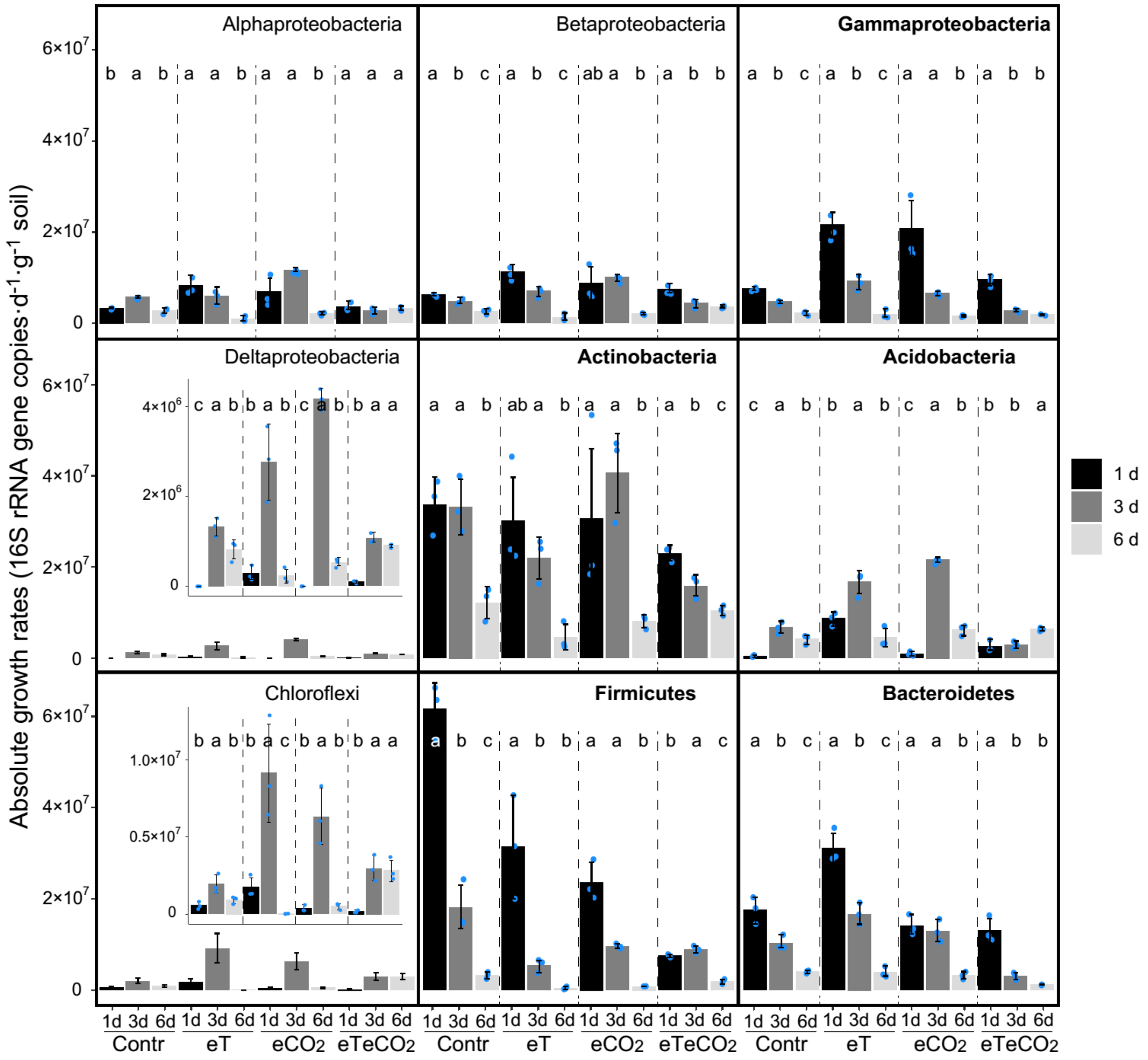

Supplementary Fig. 4

Cumulative population growth rates of dominant phyla or classes. Data are presented as mean values ± SD (n = 3 biologically independent samples). ANOVA with two-sided multiple comparisons was used for the statistical analysis. Different letters: significant differences among treatments ($p < 0.05$). For the phyla Deltaproteobacteria and Chloroflexi (relatively low growth rates), the smaller bar plots with smaller axis orders of magnitude were inserted to better highlight the variation of growth rates among treatments. Bold: the top five phyla with relatively high growth rates. Contr represents the ambient temperature and CO_2 concentration; eT represents warming only; eCO_2 represents elevated CO_2 concentration only; and $eTeCO_2$ represents combined warming and CO_2 enrichment.

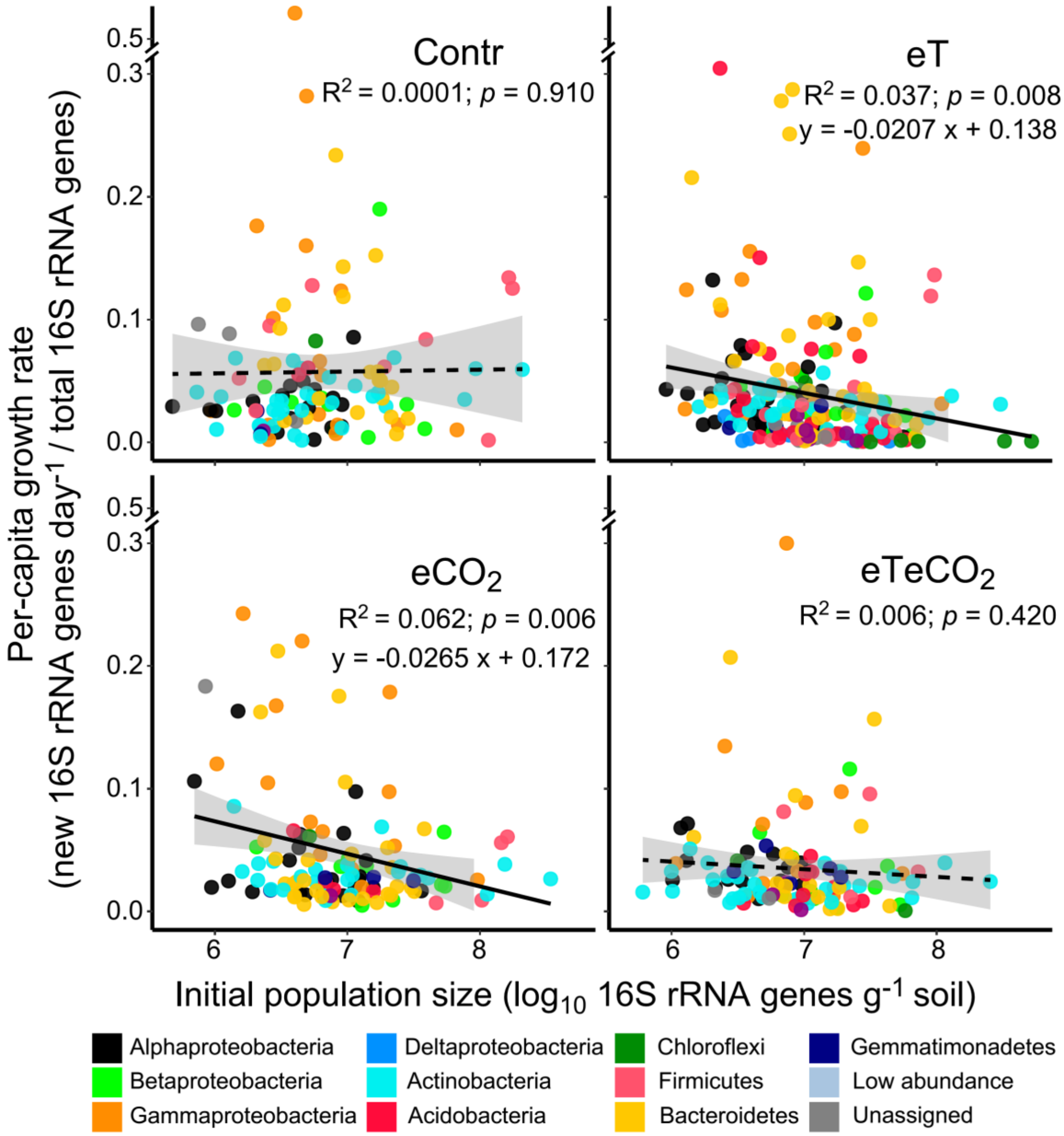

Supplementary Fig. 5

Relationship between per capita growth rates and population densities under climate change treatments. Per capita growth rates as a function of organism density for each taxonomic group during the 0-1 d time interval. Initial population size is the number of copies of the 16S gene for a given taxon at 0 d, per g soil. Per capita growth rate is growth rate of taxon-specific 16S per capita genes per day per g soil (as determined by the qSIP pipeline), normalized by taxon-specific abundance at 0 d. Initial population size is log-transformed (base 10). Linear regression was performed to explore the relationship between per capita growth rates and population densities. Solid and dashed lines denote statistically significant ($p < 0.05$) and nonsignificant ($p > 0.05$) relationships, respectively. Shade areas refer to 95% confidence interval. Contr represents the ambient temperature and CO_2 concentration; eT represents warming only; eCO_2 represents elevated CO_2 concentration only; and $eTeCO_2$ represents combined warming and CO_2 enrichment.

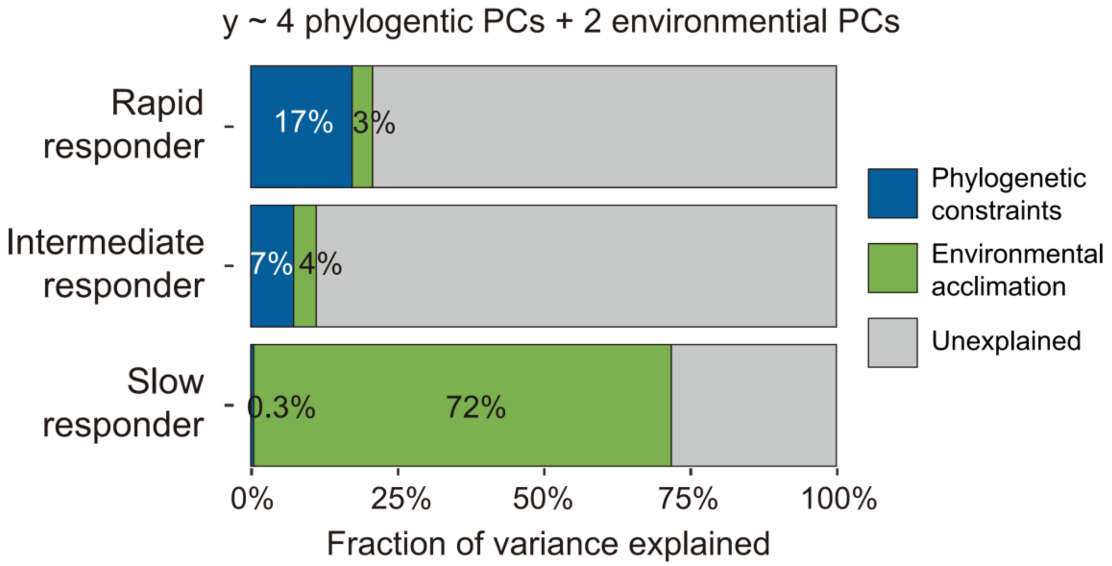

Supplementary Fig. 6

Impact of phylogenetic inertia and environment on the distribution patterns of the three pre-defined growth strategies. Horizontally stacked charts present the fraction of variance explained by phylogenetic constraints and environmental acclimation represented by their principal components (PCs).

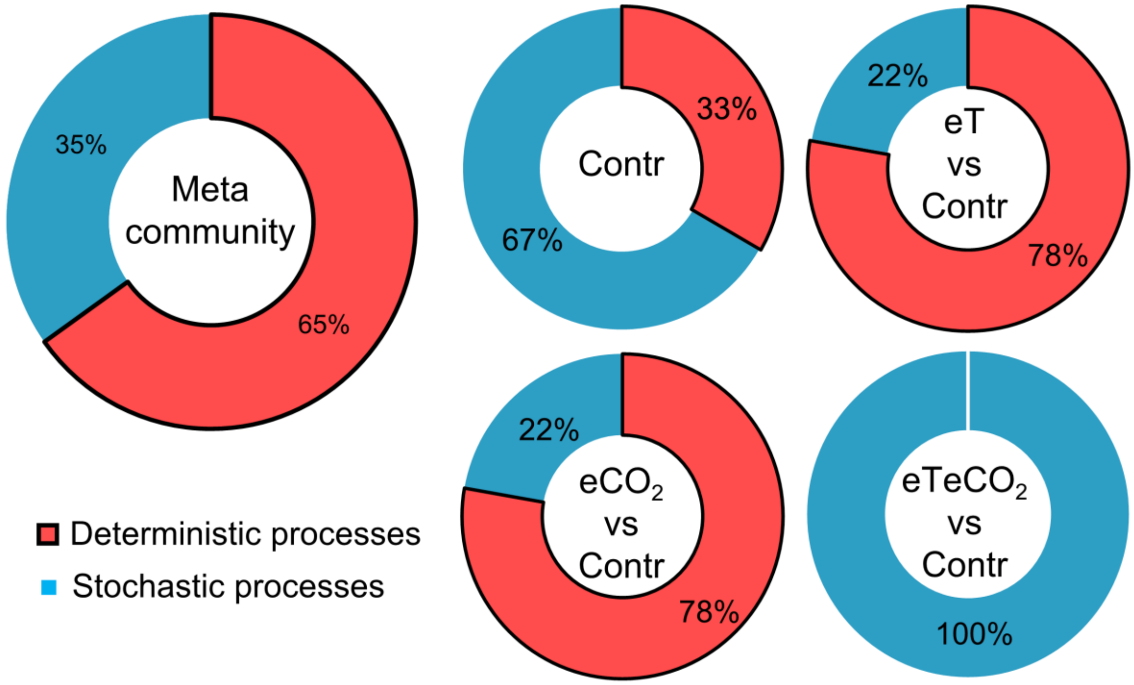

Supplementary Fig. 7

Contributions of deterministic and stochastic processes governing bacterial community assembly. The 'Metacommunity' represents the contributions of deterministic and stochastic processes on the whole community (including all four climate treatments); Contr: ambient treatment; "eT vs Contr": the community assembly processes after warming by comparing warming only with ambient treatment. The concepts of "eCO_2 vs Contr" and "$eTeCO_2$ vs Contr" are similar to that of "eT vs Contr". The deterministic processes included the processes of homogenous and heterogeneous selection; The stochastic processes included here are homogenizing dispersal, dispersal limitation, and drift. Contr represents the ambient temperature and CO_2 concentration; eT represents warming only; eCO_2 represents elevated CO_2 concentration only; and $eTeCO_2$ represents combined warming and CO_2 enrichment.
