# Supplementary material for: Elevated temperature and CO2 strongly affect the growth strategies of soil bacteria
Source: Nat Commun. 2023 Jan 24;14:391. doi: 10.1038/s41467-023-36086-y (PMC9873651; doi:10.1038/s41467-023-36086-y)
Supplement: Supplementary file 3 — Description of Additional Supplementary Files [file 41467_2023_36086_MOESM3_ESM.pdf]

Description of Additional Supplementary Files

File Name: Supplementary Data 1
Description: The data of growth rates for 1017 OTUs.

File Name: Supplementary Data 2
Description: The company names and catalog numbers of the reagents and consumables for qSIP experiment.
