# Supplementary material for: Elevated temperature and CO2 strongly affect the growth strategies of soil bacteria
Source: Nat Commun. 2023 Jan 24;14:391. doi: 10.1038/s41467-023-36086-y (PMC9873651; doi:10.1038/s41467-023-36086-y)
Supplement: Supplementary file 6 — Reporting Summary [file 41467_2023_36086_MOESM6_ESM.pdf]

nature portfolio

Corresponding author(s): Ning Ling

Last updated by author(s): Dec 31, 2022

Reporting Summary

Nature Portfolio wishes to improve the reproducibility of the work that we publish. This form provides structure for consistency and transparency in reporting. For further information on Nature Portfolio policies, see our Editorial Policies and the Editorial Policy Checklist.

Statistics

For all statistical analyses, confirm that the following items are present in the figure legend, table legend, main text, or Methods section.

n/a	Confirmed	
☐	☒	The exact sample size (*n*) for each experimental group/condition, given as a discrete number and unit of measurement
☐	☒	A statement on whether measurements were taken from distinct samples or whether the same sample was measured repeatedly
☐	☒	The statistical test(s) used AND whether they are one- or two-sided<br>*Only common tests should be described solely by name; describe more complex techniques in the Methods section.*
☒	☐	A description of all covariates tested
☐	☒	A description of any assumptions or corrections, such as tests of normality and adjustment for multiple comparisons
☐	☒	A full description of the statistical parameters including central tendency (e.g. means) or other basic estimates (e.g. regression coefficient) AND variation (e.g. standard deviation) or associated estimates of uncertainty (e.g. confidence intervals)
☐	☒	For null hypothesis testing, the test statistic (e.g. *F*, *t*, *r*) with confidence intervals, effect sizes, degrees of freedom and *P* value noted<br>*Give P values as exact values whenever suitable.*
☒	☐	For Bayesian analysis, information on the choice of priors and Markov chain Monte Carlo settings
☒	☐	For hierarchical and complex designs, identification of the appropriate level for tests and full reporting of outcomes
☐	☒	Estimates of effect sizes (e.g. Cohen's *d*, Pearson's *r*), indicating how they were calculated

Our web collection on statistics for biologists contains articles on many of the points above.

Software and code

Policy information about availability of computer code

Data collection	USEARCH (v.11.0); qSIP package, caper package, phylosignal package, picante package, FactoMineR package, car package,and vegan package in R (3.6.2); IBM SPSS Statistics 19
Data analysis	Raw sequence data generated by Illumina MiSeq sequencing were processed using USEARCH (version 11.0). Data and statistical analyses were conducted in R (version 3.6.2) and IBM SPSS Statistics 19.<br>All code for data analyses have been made publicly available on the author's Github page (https://github.com/2018203025/R-code-for-qSIP.git).

For manuscripts utilizing custom algorithms or software that are central to the research but not yet described in published literature, software must be made available to editors and reviewers. We strongly encourage code deposition in a community repository (e.g. GitHub). See the Nature Portfolio guidelines for submitting code & software for further information.

Data

Policy information about availability of data

All manuscripts must include a data availability statement. This statement should provide the following information, where applicable:

- Accession codes, unique identifiers, or web links for publicly available datasets
- A description of any restrictions on data availability
- For clinical datasets or third party data, please ensure that the statement adheres to our policy

The sequence data generated in this study have been deposited in the National Genomics Data Center (NGDC) Genome Sequence Archive (GSA) under accession code CRA006507 [https://bigd.big.ac.cn/gsa/browse/CRA006507].

Human research participants

Policy information about studies involving human research participants and Sex and Gender in Research.

Reporting on sex and gender	Not applicable.
Population characteristics	Not applicable.
Recruitment	Not applicable.
Ethics oversight	Not applicable.

Note that full information on the approval of the study protocol must also be provided in the manuscript.

Field-specific reporting

Please select the one below that is the best fit for your research. If you are not sure, read the appropriate sections before making your selection.

☐ Life sciences ☐ Behavioural & social sciences ☒ Ecological, evolutionary & environmental sciences

For a reference copy of the document with all sections, see nature.com/documents/nr-reporting-summary-flat.pdf

Ecological, evolutionary & environmental sciences study design

All studies must disclose on these points even when the disclosure is negative.

Study description	18O quantitative stable isotope probing (18O-qSIP) was used to determine microbial population-specific growth rates in soils subjected to a free-air CO2 enrichment (FACE) combined with warming. Approximately 60 g fresh soil samples were sieved through a 2-mm sieve and air-dried for 24 h at room temperature. Then, triplicate samples of dry soils (2.00 g) were incubated in the dark at room temperature in sterile plastic aerobic culture tubes (17 × 100 mm) with 400 µl of 98 atom% 18O-water or natural abundance water for 6 days, with harvests at four time points (T = 0, 1, 3, 6 d after water addition). Collectively, the qSIP experiment including three experiment factors: two types of water (16O or 18O), four climate scenarios, and four incubation time points. At each harvest, soils were destructively sampled and total soil DNA was extracted immediately using the FastDNA™ SPIN Kit for Soil. A combination of buoyant density fractionation and 16S amplicon sequencing allowed for the estimation of microbial population-specific growth rates at different sampling time.
Research sample	The soil samples using in qSIP incubation were collected from the experimental field station that simulates atmospheric CO2 enrichment and warming. The experimental site is located at Kangbo village (31°30′N, 120°33′E), Guli Township, Changshu municipality in Jiangsu Province, China, and the experiment started in 2010. Four simulated climate change treatments in this FACE system included ambient CO2 concentration and temperature as control (Contr), elevated temperature of canopy air by +2 ℃ (eT), elevated CO2 concentration up to 500 ppm (eCO2), and combined CO2 enrichment and warming treatment (eTeCO2). Each treatment was replicated in three rings with the same infrastructure. In June 2020, soil samples were collected in each climate change treatment. Ten intact topsoils (0-15 cm) samples of each plot were collected at the same time and thoroughly mixed as one composite sample. A total of 12 soil samples (4 climate treatments × 3 replicates) were shipped to the laboratory.
Sampling strategy	Sample size and incubation times in this study were based on previous qSIP experiment (Blazewicz et al. 2020 Taxon-specific microbial growth and mortality patterns reveal distinct temporal population responses to rewetting in a California grassland soil. The ISME Journal 14, 1520–1532). Each soil sample (2.00 g) was given 400 µl of 98 atom% 18O-water (18O treatment) or natural abundance water (16O treatment) to determine the isotope uptake in microbial DNA and further growth rates. Along 6 d incubation, four sampling time-points (T = 0, 1, 3, 6 d after water addition) were selected for determining average growth rates of microbial populations within 1 day, 3 days and 6 days. At each harvest, soils were destructively sampled with sterilized spoon and total soil DNA was extracted immediately using the FastDNA™ SPIN Kit for Soil. Each soil DNA sample (sampled at 1 d, 3 d and 6 d) was separated into 20 fractions after isopycnic centrifugation. The DNA of day-0 samples (unfractionated) and the fractionated DNA of fractions with buoyant density between 1.695 and 1.735 g/ml were selected for 16S amplicon sequencing by using the same primers of qPCR (515F/907R). Eleven out of 20 fractions from each ultracentrifuge tube (density between 1.695 and 1.735 g/ml) were selected

	because they contained more than 99% gene copy numbers of the 20 fractions (determined by qPCR).
Data collection	Sampling and data collection performed by authors Y.R.<br>Soil DNA was extracted and used for isopycnic centrifugation. After isopycnic centrifugation, the contents of each ultracentrifuge tube were separated into 20 fractions (~250μl each fraction). The buoyant density of each fraction was measured using a digital hand-held refractometer (Reichert, Inc., Buffalo, NY, USA) from 10 μl volumes, and total 16S rRNA gene copies of these DNA samples were quantified using the primers for V4-V5 regions: 515F (5′-GTG CCA GCM GCC GCG G-3′) and 907R (5′-CCG TCA ATT CMT TTR AGT TT-3′). Eleven out of 20 fractions from each ultracentrifuge tube (density between 1.695 and 1.735 g/ml) were selected. A total of 804 DNA samples (12 unfractionated DNA samples (sampled at 0 d) + 4 climate treatments × 3 replicates × 3 time-points × 2 types of water × 11 fractions) were sequenced using the NovaSeq6000 platform (same primers as qPCR).
Timing and spatial scale	The field experimental site is located at Kangbo village (31°30′N, 120°33′E), Guli Township, Changshu municipality in Jiangsu Province, China, and the field experiment started in 2010. Soil samples for qSIP incubation were collected from this experimental field station that simulates atmospheric CO2 enrichment and warming in June 1st, 2020, i.e., ~10 years after the start of climate simulation experiment. The field soil samples collected in one day. The fresh soil samples were immediately transported to the laboratory, and subsequently the qSIP incubation was performed. Along the qSIP incubation, four sampling time-points (T = 0, 1, 3, 6 d after water addition) were selected for determining average growth rates of microbial populations within 1 day, 3 days and 6 days. This experimental design and data collection were slightly adjusted according to a previous publication (Blazewicz et al. 2020 Taxon-specific microbial growth and mortality patterns reveal distinct temporal population responses to rewetting in a California grassland soil. The ISME Journal 14, 1520–1532).
Data exclusions	No data were excluded from the analyses.
Reproducibility	For future reproduction efforts, all code and data have been made available in publicly accessible repositories.
Randomization	Each replicate from each treatment was split and subject to each treatment level.
Blinding	Blinding was not done because aliquots of each field replicate (3 replicates per climate scenarios, across 4 climate scenarios, 12 total) were subject to every isotope treatment.

Did the study involve field work? ☒ Yes ☐ No

Field work, collection and transport

Field conditions	The climate type of experimental site is a subtropical monsoon climate in which the mean annual precipitation is between 1100-1200 mm and the annual average temperature is approximately 16℃. The high rainfall and temperature mainly occur from May through September. The soils are Gleyic Stagnic Anthrosols derived from clayey lacustrine deposits.
Location	The experimental site is located at Kangbo village (31°30′N, 120°33′E), Guli Township, Changshu municipality in Jiangsu Province, China.
Access & import/export	Samples were collected at a long-term simulated climate change field site with minimal disturbance to the surrounding area.
Disturbance	Disturbance was limited to the loss of ~ 2 kg of topsoil per plot (minimal disturbance).

Reporting for specific materials, systems and methods

We require information from authors about some types of materials, experimental systems and methods used in many studies. Here, indicate whether each material, system or method listed is relevant to your study. If you are not sure if a list item applies to your research, read the appropriate section before selecting a response.

Materials & experimental systems

n/a	Involved in the study
☒	☐ Antibodies
☒	☐ Eukaryotic cell lines
☒	☐ Palaeontology and archaeology
☒	☐ Animals and other organisms
☒	☐ Clinical data
☒	☐ Dual use research of concern

Methods

n/a	Involved in the study
☒	☐ ChIP-seq
☒	☐ Flow cytometry
☒	☐ MRI-based neuroimaging
